# High-resolution array copy number analyses for detection of deletion, gain, amplification and copy-neutral LOH in primary neuroblastoma tumors: Four cases of homozygous deletions of the CDKN2A gene

**DOI:** 10.1186/1471-2164-9-353

**Published:** 2008-07-29

**Authors:** Helena Carén, Jennie Erichsen, Linda Olsson, Charlotta Enerbäck, Rose-Marie Sjöberg, Jonas Abrahamsson, Per Kogner, Tommy Martinsson

**Affiliations:** 1 Department of Clinical Genetics, Institute of Biomedicine, Göteborg University, Sahlgrenska University Hospital, SE-41345 Göteborg, Sweden; 2 Department of Pediatrics, Göteborg University, The Queen Silvia Children's Hospital, SE-41685 Göteborg, Sweden; 3 Childhood Cancer Research Unit, Department of Woman and Child Health, Karolinska Institutet, Karolinska Hospital, SE-17176 Stockholm, Sweden

## Abstract

**Background:**

Neuroblastoma is a very heterogeneous pediatric tumor of the sympathetic nervous system showing clinically significant patterns of genetic alterations. Favorable tumors usually have near-triploid karyotypes with few structural rearrangements. Aggressive stage 4 tumors often have near-diploid or near-tetraploid karyotypes and structural rearrangements. Whole genome approaches for analysis of genome-wide copy number have been used to analyze chromosomal abnormalities in tumor samples. We have used array-based copy number analysis using oligonucleotide single nucleotide polymorphisms (SNP) arrays to analyze the chromosomal structure of a large number of neuroblastoma tumors of different clinical and biological subsets.

**Results:**

Ninety-two neuroblastoma tumors were analyzed with 50 K and/or 250 K SNP arrays from Affymetrix, using CNAG3.0 software. Thirty percent of the tumors harbored 1p deletion, 22% deletion of 11q, 26% had *MYCN *amplification and 45% 17q gain. Most of the tumors with 1p deletion were found among those with *MYCN *amplification. Loss of 11q was most commonly seen in tumors without *MYCN *amplification. In the case of *MYCN *amplification, two types were identified. One type displayed simple continuous amplicons; the other type harbored more complex rearrangements. *MYCN *was the only common gene in all cases with amplification. Complex amplification on chromosome 12 was detected in two tumors and three different overlapping regions of amplification were identified. Two regions with homozygous deletions, four cases with *CDKN2A *deletions in 9p and one case with deletion on 3p (the gene *RBMS3*) were also detected in the tumors.

**Conclusion:**

SNP arrays provide useful tools for high-resolution characterization of significant chromosomal rearrangements in neuroblastoma tumors. The mapping arrays from Affymetrix provide both copy number and allele-specific information at a resolution of 10–12 kb. Chromosome 9p, especially the gene *CDKN2A*, is subject to homozygous (four cases) and heterozygous deletions (five cases) in neuroblastoma tumors.

## Background

Neuroblastoma (NB) is the most common pediatric solid tumor. It arises from primitive sympathetic nervous cells and is characterized by clinical heterogeneity, including spontaneously regressing tumors, as well as aggressive malignant tumors. Common chromosomal abnormalities include partial deletion of the short arm of chromosome 1 (1p deletion) in 30–35% of NB tumors, additional genetic material from the long arm of chromosome 17 (17q gain) in more than 50%, amplification of the proto-oncogene *MYCN *(25–30%) and deletion of chromosome 11q and 14q [1-7]. Whole-genome array-based approaches to analyse genomic rearrangements and chromosomal abnormalities have been employed for a variety of tumors, including NB tumors. Initially, array comparative genomic hybridization (aCGH) was used. Different studies of NB have been conducted using bacterial artificial chromosome (BAC) arrays and custom-made cDNA arrays [8-15]. Based on these previous investigations, NB has been categorized into three major subtypes; types 1, 2A and 2B. Subtype 1 comprises favorable NB with near triploidy and a predominance of numerical gains and losses, mostly representing non-metastatic NB stages 1, 2 and 4S. Subtypes 2A and 2B are found in unfavorable widespread NB, stages 3 and 4, with 11q loss and 17q gain without *MYCN *amplification (subtype 2A) or with *MYCN *amplification often together with 1p deletions and 17q gain (subtype 2B) [16]. More recently commercially available high-density oligonucleotide based SNP arrays have been employed in whole-genome copy number analyses of human tumors. They have provided accurate and rapid identification of genome abnormalities at high resolution. A few groups have used commercial oligonucleotide arrays to analyze NB tumors [11,17]. We present a comprehensive genome-wide analysis of DNA copy number in 92 NB tumors using 50 K and/or 250 K gene chip arrays from Affymetrix.

## Results

**Figure 1:**
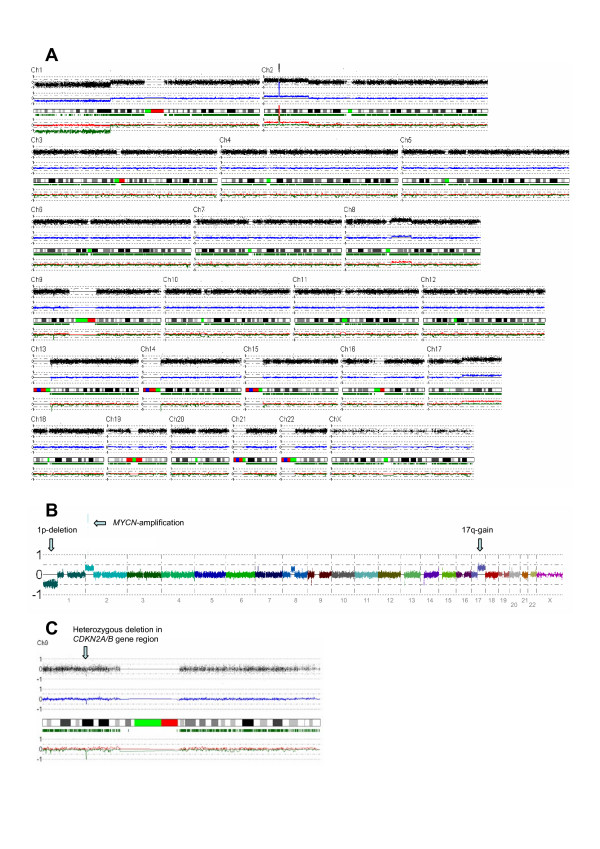
**Representative views of the technologies used**. (A) Chromosome view from the CNAG3.0 software showing a representative NB tumor. (B) 1p deletion, *MYCN *amplification and 17q gain are indicated by arrows. (C) Heterozygous deletion in chromosome 9p, in the *CDKN2A *and *CDKN2B *region.

Ninety-two NB tumors and four NB cell lines was analyzed with SNP arrays from Affymetrix. For a representative tumor, see Figure 1. Figure 1A–C shows chromosomal rearrangements analyzed with the CNAG3.0 software.

### Regions with common hemizygous deletions

#### Chromosome 1p deletion

**Figure 2:**
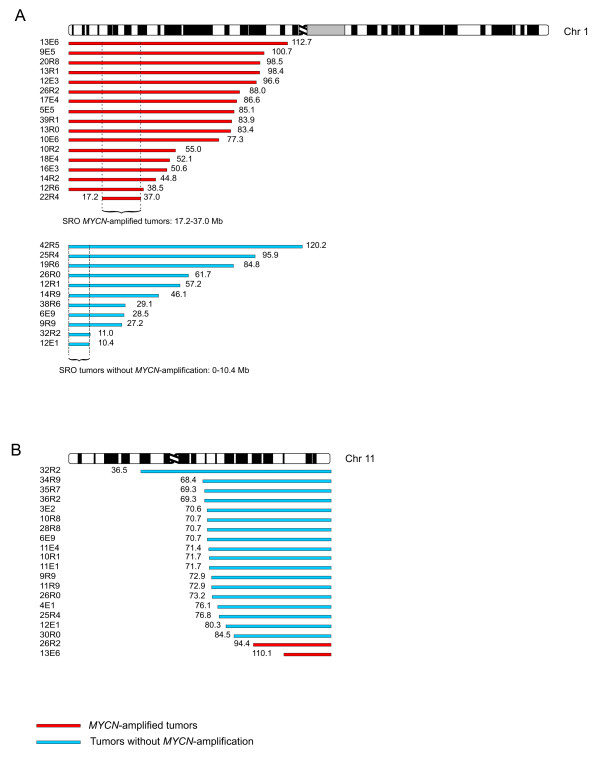
**Deletions of chromosome 1p and 11q**. Bars illustrate the deleted region; red bars for tumors with *MYCN *amplification and blue for tumors without. The positions of the breakpoints are indicated in megabases. (A) Deletions of chromosome 1p. (B) Deletions of chromosome 11q.

Loss of parts of the short arm of chromosome 1 (1p) was found in 28/92 (30%) of the tumors; 52/92 (57%) presented with intact chromosome 1. The other 12 tumors harbored other rearrangements, such as 1q gain. Seventeen of the 28 tumors with deletions also had *MYCN *amplification, whereas 11 did not (p < 2E-06). The tumors with *MYCN *amplification generally had larger 1p deletions than tumors without *MYCN *amplification (the median size of deletion for *MYCN*-amplified tumors was 84 Mb and for non-amplified 46 Mb). The five smallest deletions including the terminal of the short arm were found in tumors without amplification of the *MYCN *gene (p < 0.005), see Figure 2A. The consensus loss in the tumors with *MYCN *amplification was from position 17.2 to 37.0 Mb and, in tumors without *MYCN *amplification, from the terminal of 1p to 10.4 Mb.

#### Loss of chromosome 11q

Loss of the whole of chromosome 11 was detected in 15% (14/92) of the NB tumors. Partial loss of 11q was found in 20/92 (22%), see Figure 2B. Loss of 11q was most commonly seen in tumors without *MYCN *amplification; of the 20 tumors with 11q loss, 18 were not *MYCN *amplified. The consensus loss in the two tumors with *MYCN *amplification was 24.4 Mb (from 110.1 Mb to 134.5 Mb/qter), while it was 50 Mb (from 84.5 Mb to 134.5/qter) in tumors without *MYCN *amplification.

### Regions with homozygous and heterozygous deletions

#### Deletions in chromosome 3p

**Figure 3:**
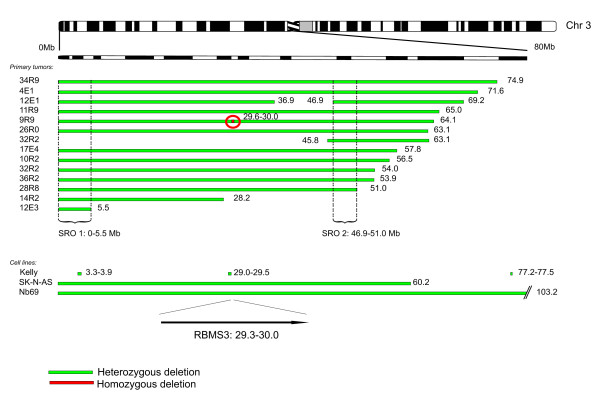
**Deletions of chromosome 3p**. Green bars illustrate heterozygous deletions and the red mark indicates a homozygous deletion in tumor 9R9 covering the gene *RBMS3*. Two regions of overlap of deletions were identified in the primary tumors.

In 9R9, a stage 3 tumor, one homozygous deletion on the short arm of chromosome 3, 29.6–30.0 Mb, was detected (Figure 3). This region contains exons 4–11 of the *RBMS3 *gene; in a patient with unfavorable outcome. In addition, 14 of 92 tumors (15%) with heterozygous deletions were detected; 12 stage 4 tumors, one stage 2 dead of disease (DOD) and the tumor mention above, see Additional file 1. Two regions with overlap of deletions were identified among these 14 tumors. The shortest region of overlap, SRO 1, located at 0–5.5 Mb, was identified in 13 of the tumors and SRO 2, from 46.9 to 51.0 Mb, in 12 of the tumors. Moreover, three cell lines had deletions of regions covering the *RBMS3 *gene. SK-N-AS and NB69 had large deletions, whereas Kelly harbored four small deletions, one of which resided in *RBMS3*. Kelly also had a homozygous deletion at region 116.7–118.5, covering the gene *LSAMP*.

#### Deletions in chromosome 9p

**Figure 4:**
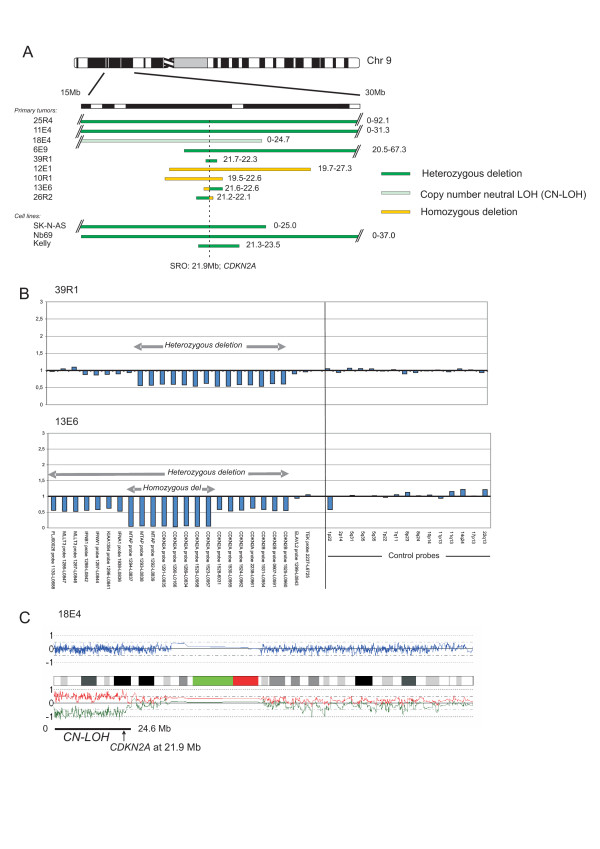
**Deletions of chromosome 9p**. (A) Array-copy number analyses of chromosome 9 deletions. Green bars illustrate heterozygous deletions, yellow bars homozygous deletions and light green CN-LOH. (B) Example of the MLPA analysis of the *CDKN2A/B *region. The SRO of deletions resides in the gene *CDKN2A*. (C) CN-LOH of 9p in tumor 18E4.

Homozygous deletions were also detected in chromosome region 9p in four NB tumors. The shortest region of overlapping deletions, at 21.9 Mb, resided in the gene *CDKN2A*. Four tumors with heterozygous deletions and one with a copy neutral loss of heterozygosity (CN-LOH) were also detected, see Figure 4A and 4C. The tumors with deletions were either high-stage NB or from patients with unfavorable outcome, see Additional file 1. The cell lines SK-N-AS, NB69 and Kelly also had heterozygous deletions in this region. In all cases, the homozygous or heterozygous losses in the *CDKN2A/B *region could be verified by multiplex ligation-dependent probe amplification (MLPA), see Figure 4B.

### Presence of copy-neutral LOH

In the total set of genome profiles from the 92 tumors, only three cases of CN-LOH could be detected; (i) the earlier mentioned case 18E4 concerning chromosome 9p (Figure 4; see above); (ii) a case of partial 5q loss in 9R9 and (iii) CN-LOH of the entire chromosome 11 in 6R9.

### Regions with amplification or gain

#### Amplification on chromosome 2p

**Figure 5:**
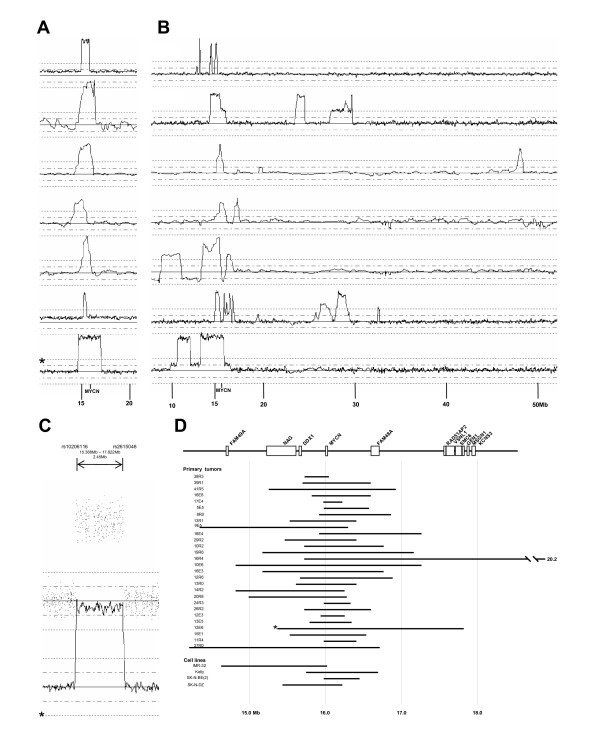
**Amplification of *MYCN***. (A) Representative tumors with simple continuous amplicons amplified. (B) Tumors with complex rearrangements. (C) The sample marked with an asterisk from the A panel in more detail. The figure shows how precisely the amplification borders can be defined using this technique. (D) The common region of amplification in tumors and cell lines. No genes other than *MYCN *were found in all cases with amplification.

Twenty-four of the 92 tumors (26%) exhibited 2p amplification without gain and 15 tumors (16%) had gains of parts of 2p without amplification, with 8/92 (9%) having both amplification and gain. Two types of *MYCN*-amplified tumor were identified. One type displayed simple amplicons, where a continuous region in and around *MYCN *was amplified. The other type harbored more complex rearrangements, where several discontinuous amplification regions were included in the amplified fragment (see Figure 5A and 5B). Apart from *MYCN*, no other genes were found to be amplified in all cases with amplification (Figure 5D).

#### Amplification on chromosome 12

**Figure 6:**
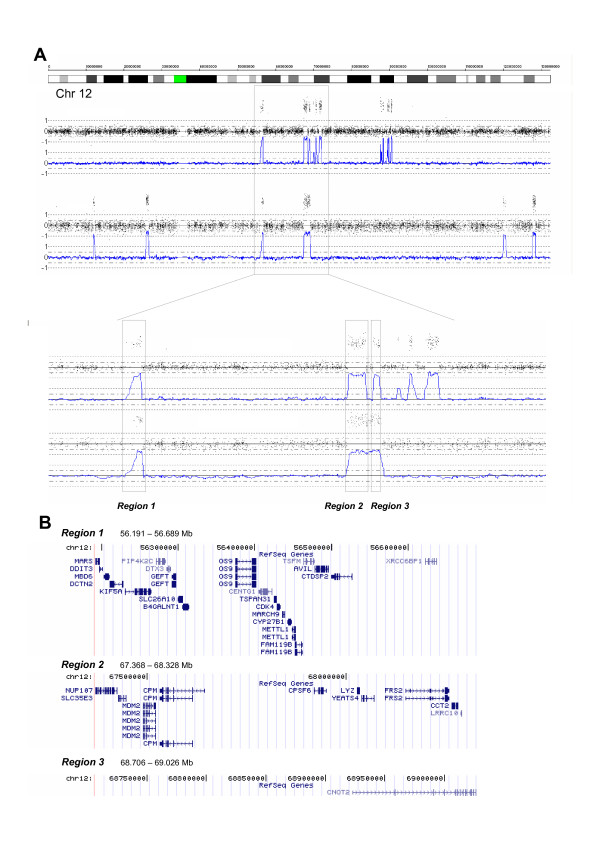
**Amplification on chromosome 12**. (A) Complex amplifications in two tumors. The common region is enhanced in the lower panel. (B) Genes located in the amplified regions.

Complex amplification on chromosome 12 was detected in two tumors, one of which also had *MYCN *amplification. Three different regions on chromosome 12 were amplified in both cases. Region I contained the genes *GLI1*, *OS9 *and *CDK4*, among others, while *MDM2 *and *YEATS4 *were two of the genes located in region II; see Figure 6 for more details.

#### Gain of chromosome 17q

**Figure 7:**
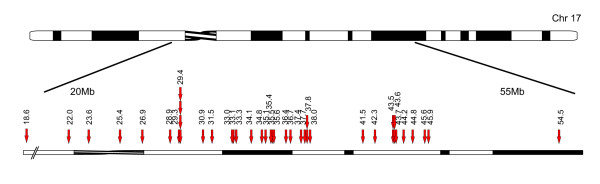
**Gain of chromosome 17q**. Arrows indicate the proximal border of the gained region which always includes the terminal of 17q. The shortest gain to be identified was located from 54.5 Mb to the terminal.

Gain of chromosome 17q was observed in 41 of 92 cases (45%). The region of gain always included the terminal of the q-arm, the smallest being 24.3 Mb (54.5–78.8/qter), see Figure 7.

Eighteen of the 92 NB tumors (20%) showed only numerical rearrangements and 16/92 tumors (17%) had no structural or numerical rearrangements on any chromosome.

## Discussion

The used technique enabled high resolution detection and mapping of all numerical and structural genomic changes in the tumor material. We could also pinpoint several previously undetected rearrangements and map them in detail. These include five new cases of homozygous deletion, which is only infrequently reported in primary neuroblastoma tumors (se discussion below). The technique proved to be fast, robust, reproducible and reliable and it is likely to be a valuable tool in future studies of neuroblastoma tumors, both in research and in the clinical setting.

### Regions of deletions

Thirty percent of the tumors had 1p deletion and those were significantly more often *MYCN *amplified. Tumors with *MYCN *amplification had generally larger 1p deletions than tumors without *MYCN *amplification and the five smallest deletions including the p-terminal were found in tumors without *MYCN *amplification, which confirms an earlier study [18]. So, when identifying the SRO in 1p deletions in NB, this will be delineated by the tumors without *MYCN *amplification showing the most distal breakpoints. It is possible that different sets of 1p-deleted genes are important for the biological behavior of the *MYCN *amplified and the non-*MYCN *amplified cases, respectively. The SRO of deletions in tumors without *MYCN *amplification was located from 0 to 10.4 Mb. For the non-amplified tumors, an interstitial SRO located between 17.2 Mb to 37.0 Mb (covering 19.8 Mb) was defined. It has previously been reported that tumors with *MYCN *amplification have 1p deletions extending proximal to 1p36 whereas non-amplified tumors more often have small terminal deletions of 1p36 [19]. Many groups have previously tried to narrow down the shortest region of overlap of deletions on chromosome 1p [20-30]. In the largest study [31], the smallest region of consistent deletion (SRD) in all but one NB tumor was located between 5.3 Mb and 6.1 Mb which resides inside our SRO for tumors without *MYCN *amplification.

There was a significant inverse correlation between 11q loss and the amplification of *MYCN*. Only 2 of the 20 tumors with a loss of chromosome 11 had *MYCN *amplification. The smallest region on chromosome 11q that was lost was detected in the two tumors that also had *MYCN *amplification (Figure 2B), the smallest being 24.4 Mb, from 110.1 Mb to 134.5/qter. The SRO in the tumors without *MYCN *amplification was defined as being 50 Mb, from 84.5 Mb to 134.5/qter. The fact that 11q deletions occurs predominantly in tumors without *MYCN *amplification is in agreement with previous studies [15,32].

Homozygous deletions are rare events in primary NB tumors. Only a few have been reported in single cases; the deletion or homozygous gene inactivation of *NF1 *[26,33], the deletion of *CDKN2A *[34], *PTEN *and *DMBDT1 *[35]. In addition, homozygous deletions in chromosome regions 1p36 [36], 3p22.3 [8] and 2q33 (*CASP8*) [37] have been detected in NB cell lines. In our material, we detected homozygous deletions in the *CDKN2A *gene in chromosome 9p21 in four tumors. This region is frequently deleted in a wide range of malignancies [38]. *CDKN2A *encodes the transcripts p16^INK4a ^and p14^ARF ^in alternative reading frames. p16^INK4a ^is an inhibitor to the cell cycle activators CDK4 and CDK6, which inactivate the tumor suppressor protein pRB, and p14^ARF ^binds and inactivates MDM2, which is responsible for the degradation of TP53, thereby leading to the stabilization of TP53 (for a review, see Sharpless et al. [39]). A second region of homozygous deletion was discovered in one NB tumor, located in chromosome region 3p24.1, harboring the gene *RBMS3*. The protein encoded by this gene is a member of a protein family which binds single-stranded DNA/RNA. We also detected two homozygous deletions in the NB cell line Kelly, one in chromosome 3p, covering the gene *LSAMP*, and one in the gene *PTPRD *in chromosome 9p. LSAMP is a neuronal surface glycoprotein that has been identified as a putative tumor suppressor gene in ovarian and renal carcinomas [40,41], also reported to be diminished in Kelly and SK-N-AS by Stallings et al. [42]. *PTPRD *is a candidate tumor suppressor gene that encodes a receptor type protein tyrosine phosphatase. This confirms the finding by Stallings et al. who have previously reported that this gene is heterozygously deleted in some NB tumors and cell lines, as well as being homozygously deleted in Kelly [42]. We identified two regions of SRO on chromosome 3p in our material; SRO 1 from 0–5.5 Mb and SRO 2 from 46.9–51.0. Our SRO 2 region overlaps one of the three SROs previously defined by Hoebeeck et al [43]. This region contains among others *RASSF1A *and *ZMYND10*; two candidate tumor suppressor genes that are epigenetically silenced in a proportion of NB tumors [44,45].

Although differences in dosage between the different alleles is very common in NB tumors, given that several tumors are in the triploid range, it is noteworthy that in the present investigation, only three of the 92 analyzed NBs showed a CN-LOH.

### Regions of gain and amplification

The most common chromosomal abnormality found in our material was the gain of chromosome 17q, found in 45% of the tumors. The SRO of gains was located from 54.5 Mb to the terminal of the long arm, including the gene *PPM1D *located at 56.0 Mb. *PPM1D *has been reported to be the most likely target of the 17q23 gain in NB tumors [46]; this gene was included in the gained region in all our tumors with 17q gain. Recently, Vandesompele and coworkers proposed that 17q gains may target two segments with one large from 44.3 Mb, in cases with a single region of gain, and one region of superimposed gains located more distally around 60 Mb [47].

The amplification of chromosome 12 was detected in two NB tumors. The gene *MDM2 *is located in the amplified region. The overexpression of *MDM2 *can result in the excessive inactivation of TP53, thereby diminishing its tumor suppressor function. Another gene is *YEATS4 *(*GAS41*; glioma amplified sequence), which has high expression in the human brain and is frequently amplified in gliomas [48]. Genes in this region have also previously been found to be amplified in single NB tumor samples or cell lines [15,49,50].

TP53 is inactivated by mutations in approximately half of all human tumors, and is believed to be abrogated in most tumors [51], although *TP53 *mutations are rare in neuroblastoma tumors [52-55]. However, mechanisms other than *TP53 *mutations could prevent TP53 activation. Previous studies have shown that silencing *CDKN2A *by methylation or the deletion or amplification of *MDM2 *are mechanisms that are responsible for inactivating TP53 in human tumors [56-59]. *PPM1D *has also been reported as a candidate proto-oncogene that may be involved in tumorigenesis through the inactivation of TP53 [51]. In our study, we detected homozygous and heterozygous deletions of *CDKN2A *and the amplification of *MDM2 *and copy number gain of *PPM1D*, which shows that these genes can be involved in the initiation/progression of neuroblastoma through the inactivation of TP53.

The fact that 17% of the NB tumors presented with no rearrangements was probably due to the tumors not having any rearrangements that could be visualized with the arrays. However, the possibility that some of these tumor samples contained regions of normal stoma cells, in spite of our efforts to obtain pure tumor material for the studies, cannot be ruled out.

## Conclusion

We have used oligonuceotide SNP arrays from Affymetrix to perform copy number analysis on chromosomal rearrangements in 92 primary NB tumors and four cell lines. The arrays, in combination with the CNAG software for analyses and visualization, make the technique very useful for analyses of tumor tissue. The mapping arrays provide both copy number and allele-specific information and have the capacity to detect duplications, amplifications, homozygous and hemizygous deletions and copy neutral LOH (genomic regions that have a normal gene copy number, albeit both gene copies originate from the same parental chromosome, i.e. uniparental disomy).

The most common structural abnormality in the tumors was the gain of 17q, which was identified in 45% of tumors, while 30% of the tumors harbored 1p deletion. Two regions of 1p-SRO deletions were identified, one larger for the tumors with *MYCN *amplification (17.2–37.0 Mb) and one smaller for those without (0–10.4 Mb). Most of the tumors with 1p deletion did also show *MYCN *amplification. Twenty-three percent of tumors had a loss of 11q; a feature most commonly seen in tumors without *MYCN *amplification. The smallest 11q deletions were found in the few tumors with amplification of *MYCN *(SRO of deletions from 110.1 Mb to 134.5/qter). Twenty-six percent of the NB had *MYCN *amplification. Two types of amplification were identified; one type displayed simple continuous amplicons, while the other type harbored more complex rearrangements. *MYCN *was the only common gene in all cases with amplification. Complex amplification on chromosome 12 was detected in two tumors and three different overlapping regions of amplification were identified. Two regions with homozygous deletions were detected indicating genes with tumor suppressor features. Four NB tumors had deletions in the *CDKN2A *gene region in 9p and one tumor had a deletion on 3p involving the *RBMS3 *gene.

## Methods

### Tumor material and DNA isolation

A panel of 92 primary NB tumors, 14 stage 1, eight stage 2, 15 stage 3, 47 stage 4 and four stage 4S, was used in this study (together with 5 tumors of unknown stage), see Additional file 1. Four NB cell lines (IMR-32, Kelly, SK-N-AS and NB69) were also used. Tumor cell content of the samples was histologically assessed in adjacent tumor tissue to that used for DNA extraction. Genomic DNA was extracted with a DNeasy blood and tissue kit (Qiagen, Hilden, Germany) according to the protocol provided by the supplier.

### Microarray experiments

#### GeneChip^® ^Human Mapping 50 K and 250 K assay

The Affymetrix 50 K Array used detects ~59,000 SNPs, while the 250 K detects ~262,000. These arrays were used to perform aCGH, where the samples were compared after the run to constitutional DNA from healthy individuals *in silico*. The array experiments were performed at our lab or at AROS Applied Biotechnology AS (Aros AB, Aarhus, Denmark) according to the protocol provided by the supplier (Affymetrix, Inc., Santa Clara, CA). Briefly, total genomic DNA (250 ng) was digested with the XbaI restriction enzyme for the 50 K array and NspI for the 250 K array and ligated to adaptors. After ligation, the template was subjected to PCR amplification using a generic primer that recognizes the adaptor sequence. The amplified DNA was fragmented with DNase I, labeled with biotin and hybridized to a GeneChip Human Mapping 50 K or 250 K array. The hybridized probes were washed using the Affymetrix Fluidics Station 450 and marked with streptavidin-phycoerythrin. The arrays were scanned using a confocal laser scanner, GeneChip Scanner 3000 (Affymetrix, Inc., Santa Clara, CA). Thirty-one NB tumors were analyzed with the 50 K array and 62 with the 250 K array (one tumor was analyzed with both the 50 K and the 250 K array).

### Data analysis

Primary data analysis was performed using GDAS software (Affymetrix, Inc., Santa Clara, CA), while further statistical studies were performed using CNAG (Copy Number Analyzer for Affymetrix GeneChip Mapping arrays) software, version 3.0 (GenomeLaboratory, Tokyo University, ) [60,61]. The UCSC genome browser, assembly March 2006 , was used to visualize gene regions. Fisher's exact test, 2-sided, was used for statistical analysis.

### Multiplex ligation-dependent probe amplification (MLPA)

MLPA analysis was performed using a probe mixture with 39 different probes and 5 control fragments. Twenty-one probes detect copy number changes in the *CDKN2A/2B *region at 9p21 (Salsa MLPA Kit P024B, MRC-Holland b.v., Amsterdam, the Netherlands). The analysis was performed according to the protocol provided by the supplier with some minor changes; the denaturation of the DNA was prolonged to 10 min and the polymerase mix was added while the samples were kept on ice. Briefly, 250 ng DNA in 5 μl TE was denaturated at 98°C and subsequently hybridized overnight (16 hours) with a mix of probes, each consisting of two parts that recognize adjacent target sequences. On day two, the hybridized probe parts were ligated with a thermostable ligase. After denaturation, PCR was performed with two universal PCR primers, amplifying all probe pairs in one reaction. The amplification products were separated by electrophoresis using an ABI 3730 Genetic Analyzer (Applied Biosystems, Foster City, CA).

## Authors' contributions

The project was initiated by, and tumor collection was organized by TM, PK and JA. HC performed experimental analyses, data analyses and drafted the manuscript. JE and LO conducted experimental analyses. R–MS handled the tumor samples. JA and PK provided clinical data. TM conducted data analyses, helped to draft the manuscript and coordinated the study. All the authors reviewed and approved the final manuscript.

## Competing interests

The authors declare that they have no competing interests.

## Supplementary Material

Additional file 1 Supplemental table Click here for file
